# Supplementary material for: Development of a computer-aided design software for the quantitative evaluation of aesthetic damage
Source: PLoS One. 2019 Dec 18;14(12):e0226322. doi: 10.1371/journal.pone.0226322 (PMC6919621; doi:10.1371/journal.pone.0226322)
Supplement: S1 Code site — 1. (ZIP) [file pone.0226322.s001.zip › [EESC_jr][Projeto]Relatorio_07_21/reports/templates_zip/gentelella-master/vendors/jquery-knob/index.html]

jQuery Knob demo


# jQuery Knob

Nice, downward compatible, touchable, jQuery dial.

\* implemented interactions : mouse click and wheel mouse, keyboard (on focus) and fingers (touch events)

× Disable display input

```
data-width="100"
data-displayInput=false
```

× 'cursor' mode

```
data-width="150"
data-cursor=true
data-thickness=.3
data-fgColor="#222222"
```

× Display previous value

```
data-displayPrevious=true
data-min="-100"
```

× Angle offset

```
data-angleOffset=90
data-linecap=round
```

× Angle offset and arc

```
data-fgColor="#66CC66"
data-angleOffset=-125
data-angleArc=250
data-rotation=anticlockwise
```

× 4-digit, step 0.1

```
data-step=".1"
data-min="-10000"
data-max="10000"
value="0"
data-displayPrevious=true
```

× Overloaded 'draw' method

```
    data-width="75"
    data-fgColor="#ffec03"
    data-skin="tron"
    data-thickness=".2"
    data-displayPrevious=true
```

```
    data-width="150"
    data-fgColor="#ffec03"
    data-skin="tron"
    data-thickness=".2"
    data-displayPrevious=true
```

```
    data-width="150"
    data-fgColor="#C0ffff"
    data-skin="tron"
    data-thickness=".1"
    data-angleOffset="180"
```

× Responsive

```
data-width="80%"
```

*Current div width is 30% of window width.  
Knob width is 80% of current div.  
Knob width is 80% of 30% of window width.  
Test resizing window.*

× Superpose (clock)

× Readonly

```
readonly (or data-readOnly=true)
data-thickness=".4"
data-fgColor="chartreuse"
```

× Dynamic

```
data-width="200"
```


```
data-width="50"
data-cursor=true
```

× Infinite || iPod click wheel

```
data-width="150"
data-cursor=true
data-thickness=".5"
data-fgColor="#AAAAAA"
data-bgColor="#FFFFFF"
data-displayInput="false"
+ some code
```

0

jQuery Knob is © 2012 Anthony Terrien - MIT License
